# A data set of global river networks and corresponding water resources zones divisions

**DOI:** 10.1038/s41597-019-0243-y

**Published:** 2019-10-22

**Authors:** Denghua Yan, Kun Wang, Tianling Qin, Baisha Weng, Hao Wang, Wuxia Bi, Xiangnan Li, Meng Li, Zhenyu Lv, Fang Liu, Shan He, Jun Ma, Zhenqian Shen, Jianwei Wang, Heng Bai, Zihao Man, Congwu Sun, Meiyu Liu, Xiaoqing Shi, Lanshu Jing, Ruochen Sun, Shuang Cao, Cailian Hao, Lina Wang, Mengtong Pei, Batsuren Dorjsuren, Mohammed Gedefaw, Abel Girma, Asaminew Abiyu

**Affiliations:** State Key Laboratory of Simulation and Regulation of Water Cycle in River Basin, China Institute of Water Resources and Hydropower Research (IWHR), Beijing, 100038 China

**Keywords:** Hydrology, Natural hazards

## Abstract

As basic data, the river networks and water resources zones (WRZ) are critical for planning, utilization, development, conservation and management of water resources. Currently, the river network and WRZ of world are most obtained based on digital elevation model data automatically, which are not accuracy enough, especially in plains. In addition, the WRZ code is inconsistent with the river network, hindering the efficiency of data in hydrology and water resources research. Based on the global 90-meter DEM data combined with a large number of auxiliary data, this paper proposed a series of methods for generating river network and water resources zones, and then obtained high-precision global river network and corresponding WRZs at level 1 to 4. The dataset provides generated rivers with high prevision and more accurate position, reasonable basin boundaries especially in inland and plain area, also the first set of global WRZ at level 1 to 4 with unified code. It can provide an important basis and support for reasonable use of water resources and sustainable social development in the world.

## Abstract (summary)

**Table:** 

Measurement(s)	river
Technology Type(s)	computational modeling technique
Factor Type(s)	geographic location
Sample Characteristic - Environment	freshwater river
Sample Characteristic - Location	Earth (Planet)

Machine-accessible metadata file describing the reported data: 10.6084/m9.figshare.9785243

## Background & Summary

Understanding the impact of climate change on water resource across different regions is highly dependent on hydrological model and data^1^. More accurate global river networks and catchment/sub-catchment boundaries are critical to more accurate water cycle simulation, water resource and risk assessments^2^. With the undeniable impacts of climate change and human activities, the processes and fluxes of terrestrial water cycle have undergone tremendous changes, which has had significant impacts on extreme hydrological events such as droughts and floods^3,4^, and induced a series of eco-environmental effects^5^, endangering the sustainable development of social economy and ecological environment^6^. It can be seen that the construction of a complete set of global river networks and corresponding water resources zones (WRZ) has been highly valued by the international communities, government departments and academia. Meanwhile, it has become a hot issue in current research on hydrology, water resources and climate change.

At present, scholars and institutions around the world have developed numerous hydrological spatial databases at national, continental and global scales. For example, Seaber et al. constructed the hydrological unit maps of the United States in 1987, which was adopted and affirmed by the Federal Government of the United States and the United States Geological Survey (USGS)^7^. In 1996, the Global River Network and Watershed Boundary Data Set (HRDRO 1 K), derived from the USGS’ 30 arc-second digital elevation model of the world (GTOPO30, about 1 km), has been produced by the EROS Data Center of the United States Geological Survey and the United Nations Environmental Program/Global Resources Information Database (UNEP/GRID)^8^. From 2006 to 2008, the World Wildlife Fund (WWF), the USGS, the International Centre for Tropical Agriculture (CIAT), the Nature Conservancy (TNC) and Kassel University in Germany have produced a global hydrological data and maps-based (HydroSHEDS) at multiple scales, from the 90-meter resolution data (SRTM)^9^. The “stream burning” method was employed to modify the surface elevation where only the large rivers and lakes located^10^. Based on the HydroSHEDS data and hydraulic geometry equations, Andreadis in 2013 developed a simple near-global database of bankfull widths and depths of rivers^11^. And Bernhard Lehner integrated and enhanced the HydroSHEDS with a new river network routing model (HydroROUT)^12^. In 2017, the USGS has developed a new global high-resolution hydrologic derivative database, entitled Hydrologic Derivatives for Modeling and Analysis (HDMA)^13^, based on HydroSHEDS, GMTED2010 (Global Multi-resolution Terrain Elevation Data 2010) and SRTM (Shuttle Radar Topography Mission) data.

Although a large number of river network data have been published, there are still some shortcomings in river accuracy, coding methods and so on. Firstly, the above river network and basin boundary are not accurate enough without sufficient manual verification. The main reason is that it is difficult to automatically obtain the correct digital river by using the original surface DEM data and GIS software, especially in inland and plain areas, due to the low spatial or vertical resolution of DEM and the lack of auxiliary data. Secondly, the codes of river network and its corresponding basin in these above datasets are different, or their stem-branch topology relationship is not clear, which hinders the use of data in hydrology and water resources research^14^. For example, Pfafstetter coding system is widely used as the river basin division method around the world currently^15,16^, which divides the hydrological units step-by-step from large to small and from coarse to fine. However, the Pfafstetter coding system does encode topologies using a numeric coding system but not fully refers to the tree structure of river network, which is not conducive to the subsequent calculation of river network relations when less than 9 division is applied^17–19^. Under these circumstances, the aim of this paper is to propose a series of methods for generating and coding a global high-precision river networks and corresponding WRZ at level 1 to 4.

This study describes the database entitled “A data set of global river networks and corresponding water resources zones divisions”, the SRTM DEM and the ASTER GDEM V2 data used to produce the river network and corresponding WRZ, based on our new method. The raster data were produced at 3-arcsecond resolution for most areas, except the Greenland and Antarctica. The derived streams and catchments are globally seamless and have been coded following our new coding system.

## Methods

We generated the river network and WRZ relying on the SRTM-DEM data and the ASTER GDEM V2 data with spatial resolutions of 90 m and 30 m, respectively. The SRTM-DEM data was measured by the US Space Agency (NASA), the National Imagery and Mapping Agency (NIMA) and the German and Italian space agencies. The ASTER GDEM V2 data (publicly available on ‘https://search.earthdata.nasa.gov/’) was developed by the Japanese METI and the US NASA.

**Fig. 1:**
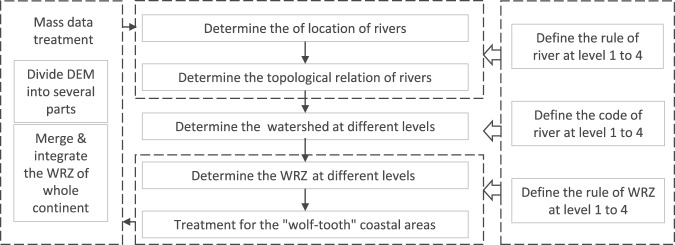
The process to build the river network and WRZ.

The methods of extracting the river network and WRZ mainly contain six parts (Fig. 1): (i) determine the location of rivers; (ii) define and code the rivers at level 1 to 4 to determine the topological relationship, hierarchical structure, and hydraulic connection of rivers; (iii) built and code the watershed at different levels to determine the river catchments in different levels, then determine the watershed and watershed boundaries; (iv) generate and code the WRZ; (v) treatment for coastal rivers with small inflow or relatively small area; (vi) mass data treatment.

### Determine the location of rivers

**Fig. 2:**
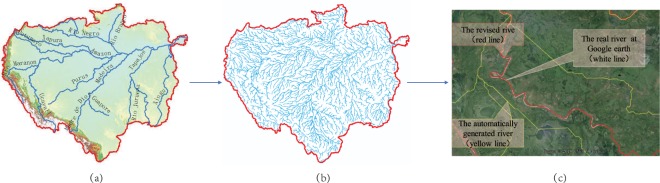
The process of revising DEM and determining the location of correct river (e.g. Amazon river Basin). (**a**) The original SRTM-DEM. (**b**) The river obtained after modifying the DEM, based on the digital rivers drawn according to the real river in Google earth. (**c**) The comparison of the rivers before and after DEM modified.

River in the mountain area could be easily generated by DEM on GIS. However, it is difficult to generate rivers in inland areas or in plain areas. For the inland area, we have developed a feasible method to obtain the river and catchment in our previous study^20^. For the plain area, we adopted and improved the “stream burning” method^21^. Specific steps include (Fig. 2):

1. Create the original river. The original rivers were firstly automatically generated based on DEM by the hydrological module of ArcGIS, through several calculation process of filling depression, flowing direction, flowing accumulation, and crating river network, similar to most studies^2^. The area threshold of rivers was judged by the National River Code of China, which stipulates that the catchment area of major rivers is larger than 1000 km^2^. The ASTER GDEM at resolution of 30 m was resampled into 90 m as same as the SRTM data before creating the river.
2. Obtain the real river from Google Earth. We firstly transformed the original river obtained automatically based the original DEM into KMZ format, and then imported it into the Google Earth software. When the Google Earth was enlarged to the finest resolution, the center line of the river was drawn according to the real river image by using the line drawing function in the Google Earth manually. In order to ensure that the drawn rivers are close to the central line in the river, a large number of the control points of the river line were drawn. Generally, one node was added per one hundred meters; while one node per fifty meters was drew for curved part of river.
3. Modify the DEM. The original DEM is modified with a gradual elevation slope based on the correct digital rivers obtained from the Google Earth using an improved “stream burning” method. A buffer around the river lines was built and modify the original DEM in order to shape a smoother transition between the original surface and the gorge. That is, we firstly calculate the maximum fill depth (Dmax) of a basin. The burning depth for rivers was Dmax, with a buffer of 3 grids around the river courses. The adjacent grid connect to the real river was reduced by 1/2 Dmax, and that of further adjacent grid was reduced by 1/4 Dmax.
4. Using the modified DEM to rebuild the river network. After revising the DEM, the correct digital river network was rebuilt by the standard hydrological processes of ArcGIS mentioned above. These rebuild rivers were converted into KML files and then revised the river accuracy according real river in Google Earth manually. The burning depth was individually adjusted, if the given elevation values significantly misrepresented the actual flow conditions in some areas.

### Determine the topological relation of rivers

To determine the topological relationship, hierarchical structure, and hydraulic connection of rivers, we established the rule of defining and coding the river network, which could clearly describe the topological relation of rivers at same or different levels.

#### Define the river level of 1 to 4

In order to unify the coding of rivers consistent with the water resources zones, the definition of river at level 1 to 4 river could be divided into two parts: in the independent WRZ and the combined WRZ (Table 1) . The Water Resources Zone in this paper refers to an area divided by a single watershed or serval neighboring watersheds, and the integrity of the watershed and water system should be maintained as much as possible^22^.

**Table 1:** Definition of rivers at level 1 to 4.

Type	Definition of the rivers at level 1 to 4
The independent WRZ	L1 river: the river that flows into the sea or lake.
	L2 river: the river that flows into the L1 river, and its confluence area is larger than one hundredth of the L1 river or 10,000 km^2^.
	L3 river: the river that flows into the L2 river, and its confluence area is larger than one hundredth of the L2 river or 1000 km^2^.
	L4 river: the river that flows into the L3 river, and its confluence area is large than one hundredth of the L3 river or 100 km^2^.
The combined WRZ	L2 river: the river which has second-order tributary.
	L3 river: the river which has first-order tributary.
	L4 river: the river which has no tributary.

**Fig. 3:**
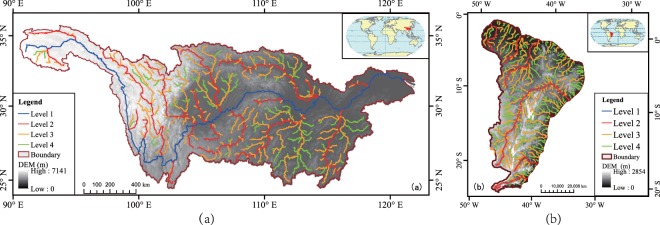
The examples of the defining the river level for two situations. The river level definition for the independent WRZ (e.g. the Yangtze River Basin in Asia, **a**) and for the combined WRZ (e.g. the Northeast part in South America, **b**).

The independent WRZ: The independent WRZ was defined as a region which includes only one independent and complete exorheic river basin, mainly the large river basins, such as the Yangtze River, the Amazon River, etc. Specific criteria for defining the rivers at level 1 to 4 are as follows (Fig. 3a):

The river at level 1 (L1 river) refers to the river that flows into the sea or lake.

The river at level 2 (L2 river) refers to the river that flows into the L1 river, and its confluence area is larger than one hundredth of the L1 river or 10,000 km^2^.

The river at level 3 (L3 river) refers to the river that flows into the L2 river, and its confluence area is larger than one hundredth of the L2 river or 1000 km^2^.

The river at level 4 (L4 river) refers to the river that flows into the L3 river, and its confluence area is large than one hundredth of the L3 river or 100 km^2^.

The tributaries that do not satisfy the above conditions were neglected.

The combined WRZ: The combined WRZ was defined as a region which contains several rivers flowing into the sea or lake, mainly small watersheds distributed in coastal areas, such as the east coast rivers in the South America. The rivers at L2, L3, and L4 levels in this combined WRZ are shown as follows (Fig. 3b).

The L2 river means the river which has second-order tributary.

The L3 river is the river which has first-order tributary.

The L4 river was defined as the river which has no tributary.

#### Code the rivers at level 1 to 4

**Fig. 4:**
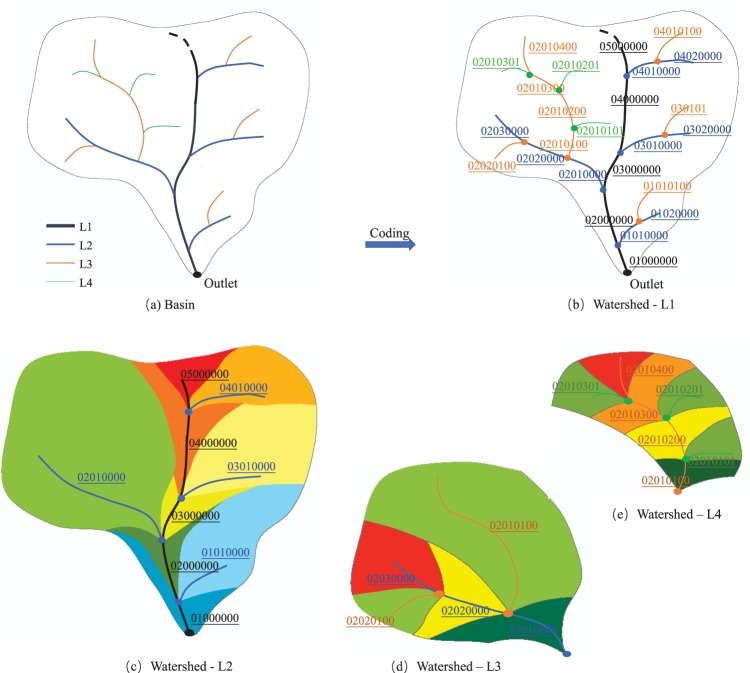
The sketch map of coding rivers and determining watershed at level 1 to 4.

The river coding method codes the river network from outlet to source, from large to small, and from coarse to fine. The schematic diagram is shown in Fig. 4. The L1 rivers (in black lines) are divided into six reaches from the outlet to the source by the L2 rivers, with code of [01000000]to [06000000], respectively. Similarly, the L2 rivers (in blue lines) are divided into several reaches by the L3 rivers, and their codes inherit the code of the L1 river section that they inflows. For example, three L2 river reaches are coded as [02010000], [02020000] and [02030000], which all inherit the code [02] of L1 river section. The L3 rivers (in orange lines) and the L4 rivers (in green lines) are coded similarly. In addition, the code is pure digital numbers, which is convenient for computer automatic coding and recognition operation. For the rare cases with multi-river confluence, the letters “A, B, C, D” are added to the river code from the second one for distinguishing, according to the cumulative amount of confluence from large to small.

#### Build the topological relation of rivers

The river coding method established in this study can identify the level and topological relationship of rivers clearly, simply and quickly. Firstly, each river reach has a code with eight digits. The first two-digits denote the reach code of L1, the second two-digits denote the reach code of L2, and so on. River topology can be identified by river code. For example, river reach [02010201] is the first tributary of L3 river reach [02010200], the second tributary of L2 river reach [02010000], the third tributary of L1 river reach [02000000]. In addition, the river reach with 01 at the end of its river code, neglecting the 0 digit, means that it is the start reach of new level. For instance, [0201000], [02010100] and [02010301] are the start river reaches of L2, L3 and L4, respectively. Moreover, water flow from river reach with large river code to small river code. For example, river reach [01000000] is the smallest river code and the outlet of this watershed. The water in all of river reaches in this watershed will flow into river reach [01000000]. This rule applies to all levels. For example, river reach [03000000] flows to [02000000] at L1, [02030000] flows to [02020000] at L2.

### Determine the watershed in different stages

Watershed is the basic unit for hydrological analysis and zoning WRZ. However, it is a challenge to determine the watershed boundaries in inland and plain area by ArcGIS automatically, due to lack of outlet, flat land, or low elevation accuracy of DEM. While for inland rivers, an innovative method was established in our previous study^20^.

For plain area, the correct river locations and corresponding watershed boundaries were obtained based on the modified DEM data above. Specifically, we used the standard GIS processing to generate watershed at different level, based on the flow direction based on the modified DEM and the selected pour points (Fig. 4c-e). We used the end point of the stem river (the outlet point of the basin) as the pour point to obtain the basin boundary (i.e., the catchment of level 1, Fig. 4b), the end point of the second level river (blue point) to obtain the watershed boundary of level 2 (i.e., the catchment of level 2, Fig. 4c), and the end point of the third level river (orange point) to obtain the watershed boundary of level 3 (i.e., the catchment of level 3, Fig. 4d), and so on. The watershed inherits the code of the corresponding river.

Our method could address some limitations of the Pfafstetter coding system mentioned above, such as maximum 9 sub-basins and low efficiency in detecting topology when less than 9 sub-basins^18^. More specific, our river coding method is based on the tree-like struct, which could divide each basin into 99 parts maximumly, and indicates a clear stem-branch topological relationship and contain more endorheic basins. For example, the river at higher level is the tributary of lower level, as shown in Fig. 4b. And the watershed code is as same as its corresponding river code, as shown in Fig. 4c-e. In addition, serval thresholds were used to control the size of sub-basin at different level, which was benefit to even the sub-basins size.

### Generate the water resources zones at level 1 to 4

**Fig. 5:**
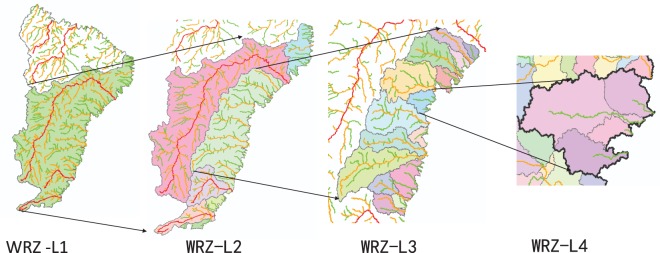
The process of zoning the combined water resources zones.

The definition of WRZ-L1 can be divided into two parts: the independent WRZ and the combined WRZ, as described above. For the former one, the L1 to L4 WRZ are their corresponding L1 to L4 basins or catchment areas (Fig. 4c-e). While it is complex for the latter one in coastal area, as shown in Fig. 5. The L1 WRZ include all the L2 to L4 WRZ within its scope. The L2 WRZ include the independent L2 river basins and all L3 river basins among them (which is defined as the L2 water resources packing zones). The L3 WRZ include the independent L3 basin and the L4 basins among them. The last L4 WRZ is the independent L4 river basin.

It is worth pointing out that the WRZ in our study is different from the Basin levels (1-12) in HydroSHEDS and the EU’s River Basin District (RBD) concept. Our new method could divide each basin into 99 sub-basins maximumly, according to the stem-branch topology, as shown in Fig. 4. By contrast, the watershed of HydroSHEDS are divided into 9 parts following the topological concept of the Pfafstetter coding system^10^. The river basin district in EU refers to the area of land and sea, made up of one or more neighboring river basins together with their associated groundwaters and coastal waters^23^. And the boundaries of river basin district in EU were obtained from different countries, without uniform data source and code number.

### Treatment for small “wolf-tooth” coastal area

Cartographic synthesis was applied for coastal rivers with small inflow or relatively small “wolf-tooth” patches, which is the coastal remaining small areas not reaching the area threshold of river of L4 (i.e. 100 km^2^). The remaining “wolf tooth” patches were obtained by cutting the continental boundaries with the integration of all the generated WRZ. Then coded these patches clockwise inheriting the code of their adjacent WRZs. Artificial coding is carried out to ensure the integrity of continental land. The continental boundaries are mainly based on that of HDMA dataset^13^, and combined with the SRTM dataset with the grid elevation equal to zero.

### Mass data treatment

We applied the method “dividing the whole into parts” to avoid exceeding the computing capacity as the DEM data of each continent is too large. As for the mass data in the process of determining the river and WRZ generated above, the combination of WRZ boundaries is essential. Oppositely, the “merging the parts into whole” method was used to recovery the WRZ boundaries of whole continent. These two opposing and complementary processes were described as below.

Dividing the DEM of continents into several parts is the first step to determine the rivers and the corresponding WRZ. Notably, the SRTM-DEM data and GTOPO 30 DEM were resampled with spatial resolution of 1 km * 1 km and merged into a global DEM data. The resampled DEM data was used to generate all of the watershed boundaries at continental scales, employing the routine hydrological processing such as filling depressions, flow direction, accumulation, and watershed in the hydrological module of ArcGIS. Secondly, the whole DEM of continent was separated into several units based on the generated watershed boundaries, including two types. The first is the individual units obtained from the large inflow basin, such as the Amazon basin, the Yangtze River basin and the Mississippi River basin. The second is the combined units that comprise several small basins among the individual basins. For example, the South America was roughly divided into seven units for processing (Fig. 2b), and the North America was divided into fifteen units.

Integrating all of the individual WRZ in each continent requires several manual processes, such as, merging boundaries, eliminating overlap, filling banks and topological inspection. On the platform of ArcGIS, we firstly merged all of the WRZ and “wolf-tooch” patch vectorial boundaries together, identified and deleted the overlapping patches manually. Then, the small banks among WRZ were filled and merged by its adjacent WRZ with similar topography and hydraulics. Finally, the topological relationship of WRZ at continent scale was examined as to remove the “overlap” or “gap” errors to obtain an accurate WRZ map.

## Data Records

**Fig. 6:**
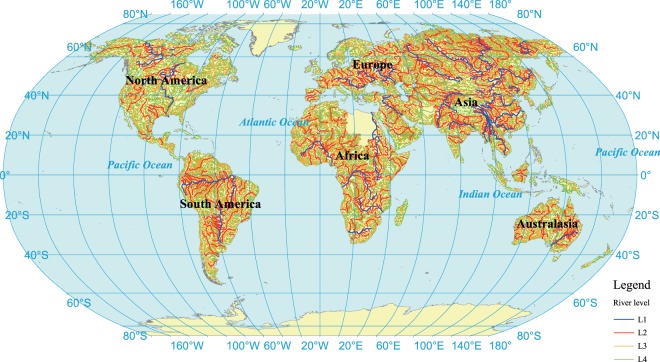
The global rivers at level 1 to 4.

**Fig. 7:**
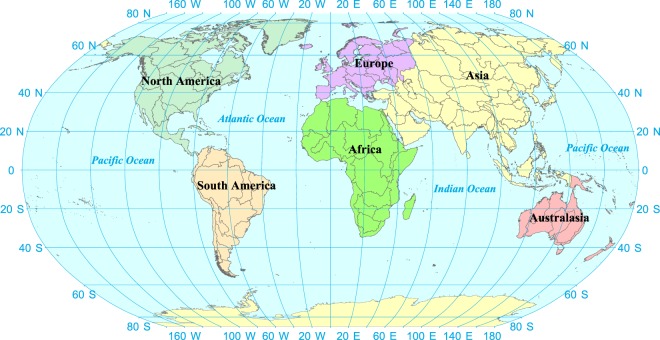
The global Water Resources Zones at level 1.

The L1 to L4 rivers networks and WRZ in the world were obtained by applying methods proposed above, which were shown in Fig. 6 and 7. The total number of global WRZ at L1 to L4 was 119, 1672, 22,871 and 60,837, respectively (Table 2). In Asia, the L1 to L4 WRZ were 41, 578, 9432 and 23,923, respectively. In Europe, there were 16, 205, 1945 and 4434 WRZ at level 1 to 4, respectively. In Africa, the number of L1 to L4 WRZ was 19, 355, 4765 and 11,624, respectively. In North America, the number of L1 to L4 WRZ was 15, 240, 2995 and 9480, respectively. The L1 to L4 WRZ in South America were 15, 220, 2929 and 7534, respectively. In Oceania, there were 13, 74, 805 and 3842 WRZ at Level 1 to 4, respectively.

**Table 2:** The distribution of Water Resources Zones in the world.

The number of WRZ	Asia	Europe	Africa	North America	South America	Australasia	Global
L1	41	16	19	15	15	13	119
L2	578	205	355	240	220	74	1672
L3	9432	1945	4765	2995	2929	805	22,871
L4	23,923	4434	11,624	9480	7534	3842	60,837

The data set are available within Figshare^24^, and is in two folders. The first folder, “Global River L1 to L4”, contains six files, which are the vector data of L1 to L4 rivers in each continent. Its attribute table mainly includes attribute information such as river ID number, river length and river coding (Table 3). The second folder, “Global Water Resources Zones L1 to L4”, contains twenty-four files, which are the vector data of L1 to L4 WRZ of each continent. Its attribute table mainly includes the ID number, code, and area of each water resources zone (Table 4). All of the shapefiles in both folders used the WGS 1984 geographic coordinates. A metadata file in ISO 19115 format^25^ is also deposited in Figshare with our data.

**Table 3:** The attribute table of rivers at level 1 to 4.

Data label	Description
FID	The ID code of river
ID	The code of river
Length	The length of river (m)

**Table 4:** The attribute table of water resources zones at level 1 to 4.

Data label	Description
FID	The ID of WRZ
ID	The code of WRZ
AREA	Area (km^2^)

## Technical Validation

The global L1 to L4 rivers obtained in this study are of high accuracy, compared with other data products, such as HydroSHEDS and HDMA river data.

### Compared with HydroSHEDS

HydroSHEDS has been developed by the Conservation Science Program of World Wildlife Fund (WWF), in partnership with the USGS, the International Centre for Tropical Agriculture (CIAT), The Nature Conservancy (TNC), and the Center for Environmental Systems Research (CESR) of the University of Kassel, Germany. HydroSHEDS is a mapping product that provides hydrographic information for regional and global-scale applications in a consistent format. HydroSHEDS is based on SRTM DEM data. However, not all of the data has been completed and released, such as the river network is at 15 arc-second resolution^26^.

Our data, overall, has better spatial resolution accuracy and clearer topology relationship than HydroSHEDS. Specifically, in our dataset, the rivers with upstream drainage area larger than 1000 km^2^ were sketched according to the real rivers in Google Earth carefully and manually, and then burned into the elevation surface to modify the original DEM and produce correct river network. The river network and corresponding watershed obtained in our study is with a resolution of 3 arc-second resolution (approx. 90 m at the equator). The watersheds and sub-basins were coded based on a new method, which made the river code correspond to its watershed code. By contrast, for the HydroSHEDS database, only large rivers and lakes were burned into the original DEM. The resolution of their released river network and watershed is at 15 arc-second (approx. 500 m at the equator). The watersheds and sub-basins were delineated and coded hierarchically based on the topological concept of the Pfafstetter coding system. The efficiency to calculate the river network relationship decreases when less than 9 divisions are applied.

### Compared with HDMA

HDMA river data, the global river data released by USGS in 2017, is currently widely used in the world and has a certain reference value, which was used for accuracy comparation^13^.

**Fig. 8:**
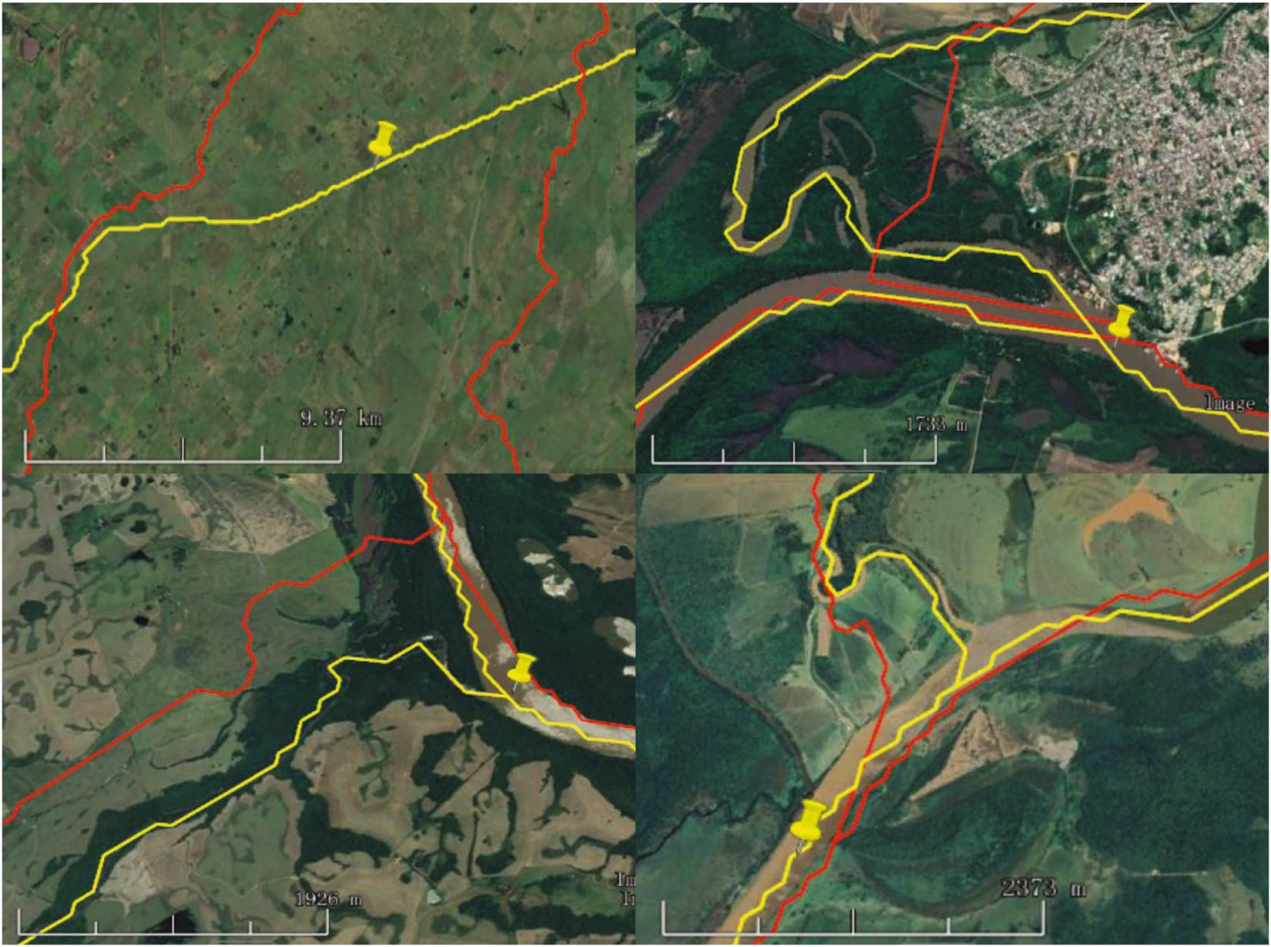
The accuracy comparison of our river and HDMA.

The specific method of accuracy comparison between our data and HDMA was carried out according to the following steps. Firstly, 400 rivers were randomly selected from L1 to L4 rivers on each continent, and each river was generated three points at random by ArcGIS, according to the basic principle of random sampling method. Secondly, after importing the above random points into Google Earth, and the center points of the real Google Earth River near to these random points were manually marked (for instance, the nail mark in Fig. 8). Thirdly, the deviation distances of these marked center real points to the rivers generated by our methods (the yellow lines in Fig. 8) and by HDMA data (the red lines in Fig. 8) were calculated and compared by ArcGIS.

It can be found in Table 5 that the average of deviation distance from the rivers generated in this study to the real river on Google Earth is 132.28 meters, while that of the HDMA river is 506.83 meters. It can be explained that the spatial resolution of DEM data used in our study is 90 m, that is to say, the river distance deviation obtained in this study is less than 2 pixels, while the HDMA river is about 6 pixels. Compared to HDMA rivers, the mean deviation distance of our L1 rivers decreased by 393.85 meters, about 69%; that of our L2 rivers decreased by 396.89 meters, about 77%; that of our L3 rivers decreased by 342.12 meters, about 76%; and L4 rivers decreased by 365.34 meters, about 74%.

**Table 5:** The accuracy comparison of different methods.

Continent	The mean deviation distance from generated rivers to the center points of real rivers (m)				
	River level	This study	HDMA	Difference	Difference in percent
Mean of all continent	1	176.73	570.58	393.85	69%
	2	119.07	515.97	396.89	77%
	3	108.13	450.24	342.12	76%
	4	125.18	490.52	365.34	74%
	Mean of all levels	132.28	506.83	374.55	74%
South America	1	65.93	866.14	800.21	92%
	2	71.41	670.75	599.34	89%
	3	55.13	326.11	270.98	83%
	4	63.86	420.24	356.38	85%
North America	1	234.33	377.33	142.99	38%
	2	106.68	411.08	304.39	74%
	3	135.54	198.22	62.67	32%
	4	98.58	243.90	145.32	60%
Europe	1	128.90	305.78	176.88	58%
	2	110.79	539.31	428.53	79%
	3	117.06	735.84	618.78	84%
	4	95.10	655.71	560.61	85%
Africa	1	364.93	935.81	570.88	61%
	2	184.89	610.68	425.79	70%
	3	102.32	668.20	565.88	85%
	4	217.21	661.89	444.68	67%
Asia	1	228.73	850.46	621.72	73%
	2	112.63	590.73	478.10	81%
	3	130.62	569.97	439.35	77%
	4	196.17	750.87	554.69	74%
Oceania	1	37.58	87.96	50.38	57%
	2	128.04	273.24	145.20	53%
	3	108.10	203.13	95.04	47%
	4	80.16	210.50	130.34	62%

As a consequence, the high-precision global river systems and WRZ at level 1 to 4 were obtained, based on the new established river coding method, the 90-meter resolution DEM data, the real river networks in the Google Earth map and numerous manual verifications. Our dataset has several advantages: (i) the generated rivers have high prevision and more accurate position, which is relatively accurate compared to other datasets, especially in inland and plain area; (ii) The WRZ were built based on the reasonable watershed and coded as same as the corresponding river code; (iii) the new river coding method could code each river segment uniquely with clear topological relationships, which overcomes some shortcomings of the existing methods. This study could provide relevant references for hydrological model calculation, water resources evaluation, drought and flood warning, and responses to global climate change. The future research could focus on the acquisition and coding of artificial channels, because the human activities, especially urbanization, have severely affected the natural river network.

## Data Availability

As mentioned above, some river and WRZ delineation steps can be programmed using serval ArcGIS standard process which was provided in our data set. These processes were packaged into an integrated model to improve the efficiency. This model, deposited in Figshare with our data named “Method-Toolbox.tbx”, contains four functions, “modify the original dem”, “generate the flow direction and accumulation”, “generate the river network” and “generate the WRZ”. Before running this, it needs to install ArcGIS 10.2 or above version in the Windows system. Then, the scripts can be opened by the ArcGIS and the corresponding dialog box will show to add the input and output files path. By finishing all the above steps, it will automatically generate all the output files, such as the “modified DEM”, “river network” and “the WRZ”.
